# Correction: Blade coated P3HT:non-fullerene acceptor solar cells: a high-throughput parameter study with a focus on up-scalability

**DOI:** 10.1039/d2ta90177c

**Published:** 2022-09-03

**Authors:** Enrique Pascual-San-José, Xabier Rodríguez-Martínez, Rana Adel-Abdelaleim, Marco Stella, Eugenia Martínez-Ferrero, Mariano Campoy-Quiles

**Affiliations:** Institut de Ciència de Materials de Barcelona (ICMAB-CSIC) Campus de la UAB, Bellaterra Barcelona 08193 Spain mcampoy@icmab.es; EURECAT, Centre Tecnológic de Catalunya, Parc Científic i de la Innovació TecnoCampus Av. de Ernest Lluch, 36, Mataró Barcelona 08302 Spain

## Abstract

Correction for ‘Blade coated P3HT:non-fullerene acceptor solar cells: a high-throughput parameter study with a focus on up-scalability’ by Enrique Pascual-San-José *et al.*, *J. Mater. Chem. A*, 2019, **7**, 20369–20382, https://doi.org/10.1039/C9TA07361B.

The authors regret that the labels for IDTBR and IDFBR in Fig. 1b were swapped and referred to the incorrect structures.

A corrected version of Fig. 1 with the correct labels for structures IDTBR and IDFBR is shown below.

**Fig. 1:**
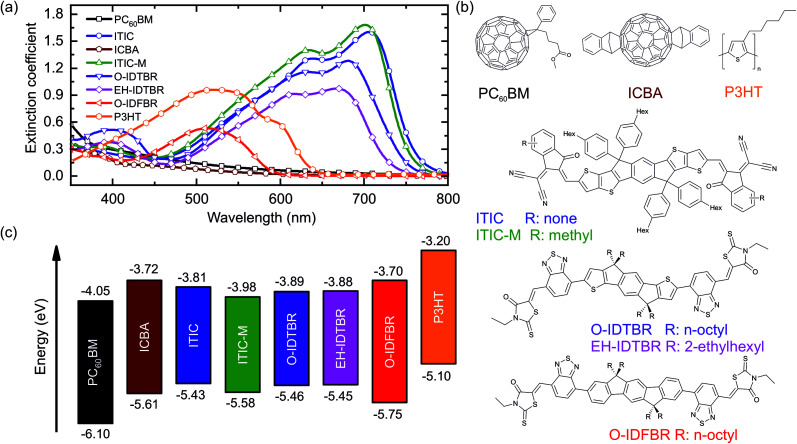
Material properties for the studied systems, namely P3HT, PC_60_BM, ICBA, ITIC, ITIC-M, O-IDTBR, EH-IDTBR, and O-IDFBR. (a) The extinction coefficient obtained by VASE, (b) chemical structures and (c) energetic levels (obtained from the literature with the corresponding references given in the main manuscript).

The Royal Society of Chemistry apologises for these errors and any consequent inconvenience to authors and readers.

## Supplementary Material

